# Death after Ether Anæsthesia

**Published:** 1890-01

**Authors:** 


Death after Ether Anæsthesia.—A case of death from ether inhalation recently occurred at Bellevue Hospital. The patient was a male, and the ether was being given by Dr. Theodore, of the house staff of the hospital, preliminary to an operation upon the throat. The coroner's jury in the case rendered a verdict that death was caused by asphyxiation due to the administration of ether complicated with cystic degeneration of the kidneys. Dr. Dunham, however, is of the opinion that death is due to heart failure, rather than asphyxiation. All the usual precautions were taken in the use of the anæsthetic.—*Boston Med. and Surg. Journal*, Nov. 14, 1889.

